# Genetic Detection of *Pseudomonas* spp*.* in Commercial Amazonian Fish

**DOI:** 10.3390/ijerph10093954

**Published:** 2013-08-29

**Authors:** Alba Ardura, Ana R. Linde, Eva Garcia-Vazquez

**Affiliations:** 1 Department of Functional Biology, University of Oviedo. C/Julian Claveria s/n, Oviedo 33006, Spain; E-Mail: egv@uniovi.es; 2 Laboratory of Toxicology, National School of Public Health, Fundaçao Oswaldo Cruz, Rio de Janeiro 21041, Brazil; E-Mail: linde14@yahoo.com

**Keywords:** Amazon River, *Pseudomonas*, molecular tests, commercial fish, food safety

## Abstract

Brazilian freshwater fish caught from large drainages like the River Amazon represent a million ton market in expansion, which is of enormous importance for export to other continents as exotic seafood. A guarantee of bacteriological safety is required for international exports that comprise a set of different bacteria but not any *Pseudomonas*. However, diarrhoea, infections and even septicaemia caused by some *Pseudomonas* species have been reported, especially in immune-depressed patients. In this work we have employed PCR-based methodology for identifying *Pseudomonas* species in commercial fish caught from two different areas within the Amazon basin. Most fish caught from the downstream tributary River Tapajòs were contaminated by five different *Pseudomonas* species. All fish samples obtained from the River Negro tributary (Manaus markets) contained *Pseudomonas*, but a less diverse community with only two species. The most dangerous *Pseudomonas* species for human health, *P. aeruginosa*, was not found and consumption of these fish (from their *Pseudomonas* content) can be considered safe for healthy consumers. As a precautionary approach we suggest considering *Pseudomonas* in routine bacteriological surveys of imported seafood.

## 1. Introduction

Brazil contains a rich biodiversity of animal and plant taxa distributed at varied latitudes, and could open a new international market for high quality food products, perhaps targeting delicatessen shops and specialized restaurants. Such a market would be exclusive of Brazil because many species from some regions, like the Amazon, are unique and endemic. Due to their enormous diversity [1,2], Amazonian fishes represent a potentially interesting sector for export. Tools for labeling these fishes are currently being developed [3], aimed at enabling Amazonian fish introduction in demanding markets like the European one, where the normatives on traceability and food security control are strict (e.g., European Directives CE-178/2002; CE-1759/2006).

Introduction of exotic species in a new market encompasses some potential risks. One of them is introduction of parasites or pathogens endemic of their native region [4]. Control of such pathogens in the importer country may not be required if they are normally absent from local food. An example applicable to the fish trade could be *Pseudomonas*. These bacteria constitute a part of the normal fish microbiota, but are opportunistic and may become infectious and spread diseases in stressed fish [5]. *Pseudomonas* can be a problem for human consumers too. They appear in processes of seafood spoilage [6,7] and in ready-to-eat products [8]. In some conditions they can become human pathogens and cause infection. Many pathogeneses of *Pseudomonas* in humans, generally caused by only one species (most frequently *P. aeruginosa*), are health-care associated illnesses [9,10], and the risk of disease by ingestion in healthy consumers has been considered generally low in developed countries [11]. However, such risk exists and can be serious depending on the circumstances. Contamination with enterotoxigenic *Pseudomonas* has been reported from food and drinking water samples in some countries [12]. Jertborn and Svennerholm [13] have discovered enterotoxin-producing *Pseudomonas* in Swedish travellers with diarrhoea, somewhat more frequently in travellers visiting Africa, Asia and Latin America. In association with other bacteria they can cause severe cholera symptoms in healthy adults [14]. Even when their enterotoxigenic activity is weak they can still produce diarrhoea in immunodeficient individuals [15]. In addition they can cause skin problems; their presence in cosmetics is considered a health threat in the U.S. [16], and have produced outbreaks of skin infections [17]. Therefore there are infection risks when manipulating contaminated seafood.

Notwithstanding the information provided above, *Pseudomonas* species are not catalogued as a foodborne pathogen in Europe and other regions. Control tests of imported fish and shellfish include various bacterial species like *Escherichia coli*, *C. botulinum*, *Listeria monocytogenes*, *Staphylococcus aureus*, *Enterobacter sakazakii* and *Salmonella* sp*.*, but not *Pseudomonas* spp*.* (e.g., European Council Directive 1991, 1995 and Council Directive 1998; Canadian Food Inspection Agency [18], European Comission [19], American Food Safety and Inspection Service (FSIS) [20].

The aim of this study was to investigate the presence of *Pseudomonas* spp*.* in samples of commercial fish sold in Brazilian markets from two different Amazonian states (Table 1): the River Tapajós (Para) and the River Negro (Amazonas). The results may inform about the convenience of including these bacteria in routine controls for Brazilian fish exports as well as in local markets. The molecular tools used in this study were PCR-amplification with *Pseudomonas*-specific primers and sequencing 16S rRNA genes. This type of methodology is highly sensitive and has been employed in other surveys of foodborne bacteria [21,22].

**Table 1:** Samples analyzed, fish species with their common names and *Pseudomonas* species identified.

Sample	Origin	Fish spp.	Common name	*Pseudomonas* species
T1	Tapajós	*Prochilodus nigricans*	Curimatá	*Pseudomonas psychrophila*
T2	Tapajós	*Cetopsis candiru*	Candiru	*Pseudomonas* spp.
T3	Tapajós	*Leporinus piau*	Piau	*Pseudomonas psychrophila*
T4	Tapajós	*Leporinus piau*	Piau	*Pseudomonas syringae*
T5	Tapajós	*Serrasalmus rhombeus*	Piranha	*Pseudomonas fragi*
T6	Tapajós	*Leporinus piau*	Piau	*Pseudomonas fluorescens*
T7	Tapajós	*Ageneiosus brevifilis*	Bocudo	*Pseudomonas fluorescens*
T8	Tapajós	*Leporinus piau*	Piau	*Pseudomonas psychrophila*
T9	Tapajós	*Leporinus piau*	Piau	*Pseudomonas syringae*
T10	Tapajós	*Leporinus piau*	Piau	*Pseudomonas fluorescens*
T11	Tapajós	*Leporinus piau*	Piau	*Pseudomonas* spp.
T12	Tapajós	*Leporinus piau*	Piau	*Pseudomonas psychrophila*
T13	Tapajós	*Prochilodus nigricans*	Curimatá	*Pseudomonas psychrophila*
T14	Tapajós	*Leporinus piau*	Piau	*Pseudomonas fragi*
T15	Tapajós	*Leporinus piau*	Piau	*Pseudomonas psychrophila*
T16	Tapajós	*Leporinus piau*	Piau	*Pseudomonas putida*
T17	Tapajós	*Leporinus piau*	Piau	-
T18	Tapajós	*Leporinus piau*	Piau	-
M1	Negro	*Chaetobranchopsis orbicularis*	Acará branco	*Pseudomonas putida*
M2	Negro	*Astonotus ocellatus*	Acará-açú	*Pseudomonas putida*
M3	Negro	*Astonotus ocellatus*	Acará-açú	*Pseudomonas putida*
M4	Negro	*Astonotus ocellatus*	Acará-açú	*Pseudomonas putida*
M5	Negro	*Osteoglossum bicirrhosum*	Aruanà	*Pseudomonas putida*
M6	Negro	*Brachypatystoma rousseauxii*	Dourada	*Pseudomonas putida*
M7	Negro	*Semaprochilodus insignis*	Jaraquí	*Pseudomonas putida*
M8	Negro	*Plagioscion squamosissimus*	Pescada	*Pseudomonas putida*
M9	Negro	*Phractocephalus hemioliopterus*	Pirarara	*Pseudomonas psychrophila*
M10	Negro	*Pseudoplatystoma fasciatum*	Surubim	*Pseudomonas putida*
M11	Negro	*Cichla temensis*	Tucunaré	*Pseudomonas putida*
M12	Negro	*Cichla temensis*	Tucunaré	*Pseudomonas putida*

## 2. Experimental Section

### 2.1. Sampling

**Figure 1:**
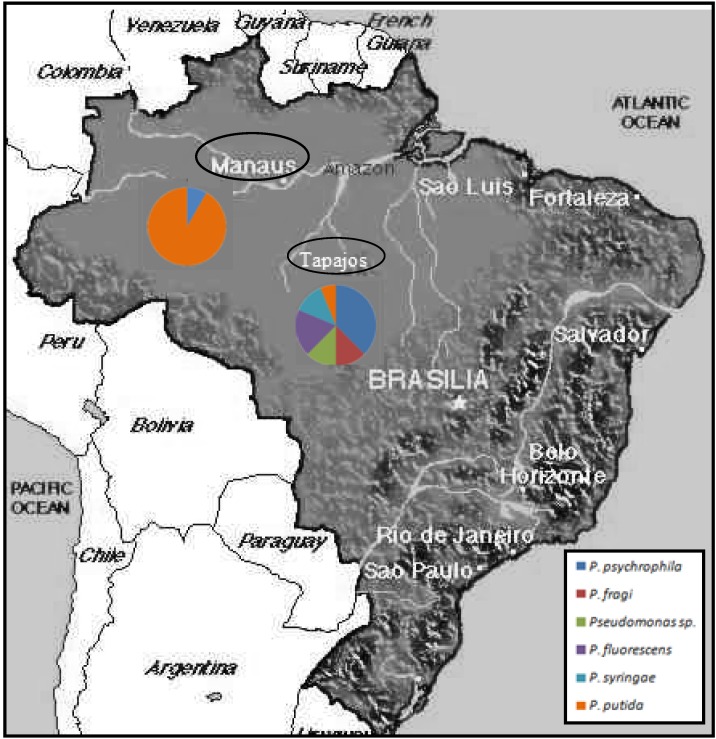
A map with proportions of different *Pseudomonas* species found in each sampling site: Manaus and Tapajós.

The 30 fish samples analyzed (Table 1) were obtained from two different tributaries within the Amazon basin (Figure 1): the River Negro (Manaus markets; n = 12) and the River Tapajós (n = 18), and were directly purchased from fishermen in local harbors and markets. All fish specimens were morphologically identified *in situ* by visual inspection and taxonomically classified employing standard taxonomic guides. After cleaning the fish surface with ethanol, samples of muscle (the edible tissue) were excised in situ with sterilized blades and tweezers and immediately stored in absolute ethanol. Ethanol-preserved samples were transported in coolers to the laboratory for genetic analysis.

### 2.2. Genetic Analyses

DNA extraction and PCR amplification were carried out in sterile conditions to prevent cross-contamination of samples during the process. Total DNA was extracted from a small piece (approximately 5 mg) of alcohol-preserved fish tissue by the standard protocol of Estoup *et al*. [23], using Chelex^®^ resin (Bio-Rad Laboratories, Hercules, CA, USA). Chelex^®^ is a chelating material used to purify other compounds from a tissue via ion exchange. It is often used for DNA extraction in preparation for PCR. Polar resin beads bind polar cellular components after breaking open cells, while DNA and RNA remain suspended in water solution above the Chelex^®^. The tissue was introduced in an Eppendorf tube with 500 µL of Chelex^®^ resin (10%) and 7 µL of Proteinase K (20 mg/mL). It was incubated at 55 °C for 90 min. The DNA was dissolved in the aqueous solution. Finally, it was introduced in an oven at 100 °C during 20 min for inactivating the enzyme. The tube was stored at 4 °C or frozen at −20 °C for long-time preservation.

A fragment of the 16S rRNA gene was amplified by polymerase chain reaction (PCR), employing the *Pseudomonas* genus specific primers PA-GS-F (5′-GACGGGTGAGTAATGCCTA-3′) and PA-GS-R (5′-CACTGGTGTTCCTTCCTATA-3′) described by Spilker *et al*. [24] They amplify a DNA region of 618 nucleotides located between the sites 113 and 712, position and size relative to 16S rDNA sequence of *Pseudomonas aeruginosa* AT2 (AB091760) [24]. The amplification reaction was performed in a total volume of 40 µL, including Promega (Madison, WI, USA) Buffer 1X, 2.5 mM MgCl_2_, 0.25 mM dNTPs, 20 pmol of each primer, 20 ng of template DNA, and 1 U of DNA Taq polymerase (Promega). The PCR conditions were the following: an initial denaturation at 95 °C for 5 min, 10 cycles at 94 °C for 15 s, annealing at 53 °C for 30 s and elongation at 72 °C for 45 s. This was repeated for 25 cycles, increasing the elongation step at 72 °C by 5 s every cycle. The final extension phase was at 72 °C for 10 min.

PCR products were visualized in 2% agarose gels with 3 µL of 10 mg/mL ethidium bromide. Stained bands were excised from the gel, and DNA was purified with an Eppendorf PerfectPrep Gel CleanUp^®^ kit prior to sequencing. After that, amplified and purified products were precipitated using standard 2-propanol precipitation and re-suspended in formamide.

Sequencing was performed in an ABI PRISM 3100 Genetic Analyzer (Applied Biosystems, Foster City, CA, USA) with BigDye 3.1 terminator system, at the Sequencing Unit of the University of Oviedo (Oviedo, Spain).

### 2.3. Sequence Edition and Phylogenetic Analysis

Sequences obtained from the 16S rRNA gene amplicons were visualized and edited employing the BioEdit Sequence Alignment Editor software [25]. Sequences were aligned with the ClustalW application [26] included in BioEdit.

The phylogenetic analysis was performed with the software MEGA 4.0 [27]. This software was employed to reconstruct the phylogenetic trees of the *Pseudomonas* species found in fish samples from 16S rDNA sequences. The methodology chosen was the neighbor-joining (NJ), the standard method of phylogenetic inference in DNA barcoding studies [28] because it allows to rapid analysis of large species assemblages [29]. The molecular substitution model was chosen using the software jModeltest [30] to determine the best suited model of sequence evolution and accompanying evolutionary parameter values for the data. Robustness of the NJ topology was assessed using 2,000 bootstrap replicates.

*Pseudomonas* species identification was made by comparing generated 16S rDNA sequences with reference sequences present in the GenBank database by means of BLAST online program [31].

### 2.4. Pseudomonas Diversity Estimates

*Pseudomonas* species diversity in each Amazonian location was estimated by means of ecological index (Shannon, H) using PRIMER 6 (Software package from the Plymouth Marine Laboratory, Lutton, Ivybridge, UK). The number of haplotypes (h) and nucleotide diversity (π) were calculated with the ARLEQUIN software [32,33].

### 2.5. Statistics

To compare the proportion of contaminated fish between locations, chi-square statistics was employed. Analysis was carried out using the SPSS 13.0 software (SPSS Inc., Chicago, IL, USA).

## 3. Results and Discussion

**Figure 2:**
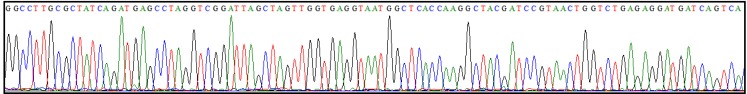
Chromatogram of a DNA sequence corresponding to the 16S rRNA gene fragment of a *Pseudomonas putida* (T16) found in *Leporinus piau* from Tapajós.

Positive PCR amplification was obtained with *Pseudomonas* specific primers for a fragment of the 16S rDNA [24] from 28 Amazonian fish out of 30 samples analyzed (93.3%): 16 from the River Tapajòs and 12 from the River Negro. Cross-contamination of samples during the process of DNA analysis can be reasonably excluded since the two samples from Tapajòs that did not provide positive PCR amplification (Table 1) could be considered *Pseudomonas*-free. Sequences were very clean (an example is in Figure 2) and mixture of species was not found for any sample. This does not exclude their presence but indicates that, if other *Pseudomonas* were present in a sample, they were likely in a lower concentration; the PCR primers would anneal preferentially with the most abundant target DNA. The sequences are available in the GenBank public database [31] under the accession numbers JF745541-JF745568.

The 16S rDNA sequences obtained allowed to identifying five *Pseudomonas* species in Tapajós (Table 1): *Pseudomonas pshychrophila*, *P. fragi*, *P. fluorescens*, *P. syringae* and *P. putida*, based on 100% of similarity with other reference sequences of those species included in the GenBank. Two fish contained *Pseudomonas* but the species could not be identified because the alignment obtained did not yield 100% similarity with any other *Pseudomonas* species included in the GenBank, therefore they were classed as *Pseudomonas* sp. On the other hand, Manaus fish samples carried only two *Pseudomonas* species: *P. putida* and *P. pshychrophila* (Table 1).

For the fish carrier, *Pseudomonas* contamination affected different fish species (Table 1), but association fish-*Pseudomonas* species could not be properly tested due to reduced number of some fish species.

**Figure 3:**
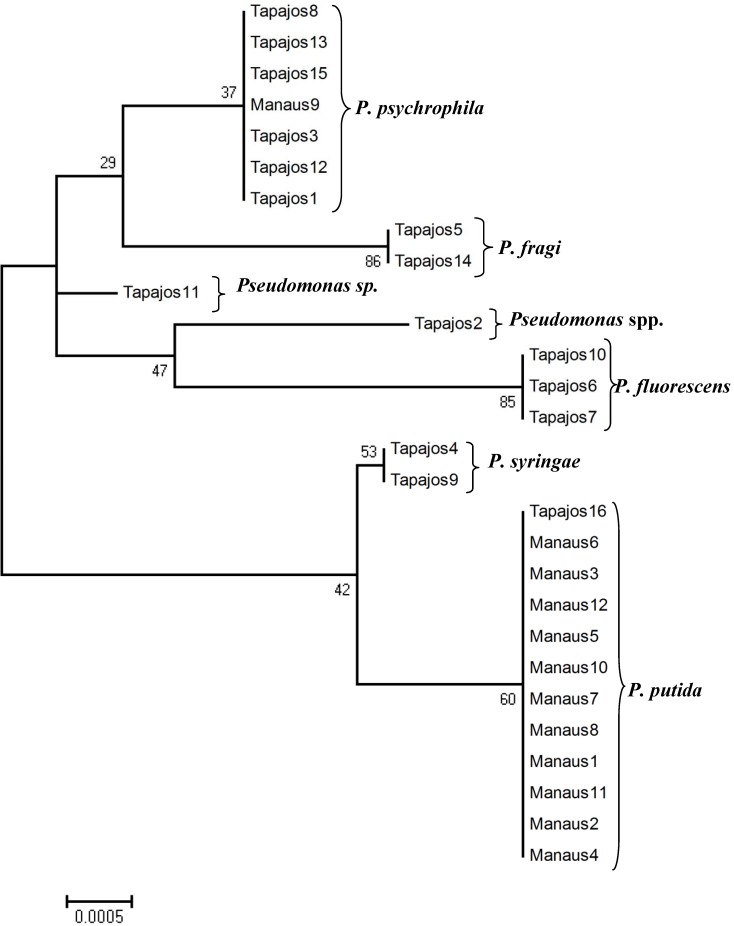
Neighbour-Joining tree constructed based on 16S rDNA *Pseudomonas* sequences found in this survey. Bootstrap values (in percent).

The *Pseudomonas* found in the two locations clustered in two main branches in a phylogenetic tree (Figure 3), supported by relatively low bootstrapping. One contained *Pseudomonas putida* and *P. syringae* and the other clustered the other three species and the unidentified sequences, which should logically correspond to species of the same group.

**Figure 4:**
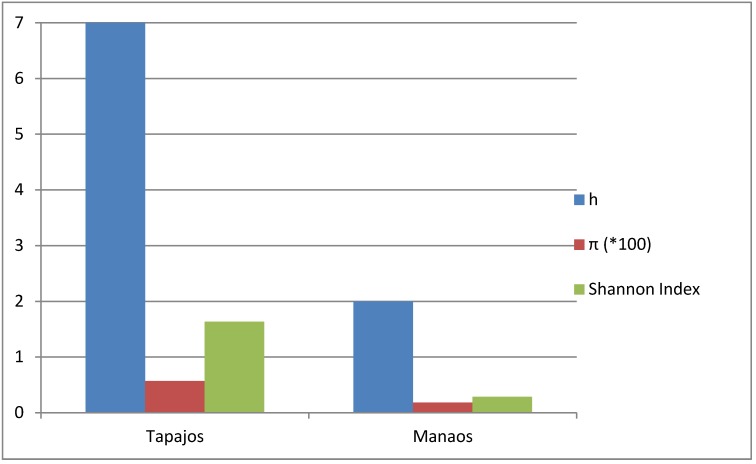
Diversity parameters of fishborne *Pseudomonas* communities from the Amazonian Tapajòs and Negro tributaries. Metagenetic h and π parameters, and Shannon index.

Although the proportion of contaminated fish was similar in the two locations analyzed, Tapajós fish samples contained more *Pseudomonas* sp*.* species and therefore higher bacterial diversity, both ecological and genetic, than Manaus commercial fish (Figure 4). The species composition of the *Pseudomonas* complex found in the two locations was significantly different (Chi-square value = 19.26, *p* < 0.001), clearly due to much higher proportion of *P. putida* and *P. psychrophila* in Manaus and Tapajós fish, respectively (Figure 1).

The results presented here, although based on small sample sizes, suggest that *Pseudomonas* are endemically present in Amazonian fish sold in local markets since most analyzed fish yielded positive PCR amplification for these bacteria. *Pseudomonas aeruginosa*, the most dangerous species for human health [9,10,17] and especially for consumers [13,15], was not detected. Therefore consumption of these fish can be considered generally safe for healthy people, at least from their *Pseudomonas* content.

The characteristics of the *Pseudomonas* species found from Brazilian fish samples (Table 2) may suggest the origin of the contamination. Fish infection in some Tapajós samples was suggested by the presence of the well-known fish pathogen *P. fluorescens*, which is considered as opportunistic pathogenic species in aquaculture [34,35], responsible for bacterial septicemia in fish. This species was present in three (16.7%) samples from Tapajós, but in none from Manaus (Figure 1). *Pseudomonas* infections in fish are promoted by different stressors [6,36]. Environmental stress produced by mining has been reported in the River Tapajós [37], and could contribute to facilitate fish infection by opportunistic *Pseudomonas*.

*P. putida* was found in most Manaus samples (Figure 1) and in only one sample from Tapajós. Different *Pseudomonas* species have been associated with seafood (including chilled fish) spoilage, for example *P. fragi* [6], therefore the likely origin of contamination of these samples could be seafood manipulation, long time of storage before selling or simply opportunistic growth of these bacteria on fish exposed without protection in the open-door local markets. *P. putida* infections have also been reported in fish species, for example in farmed rainbow trout [38], also associated to stress, therefore this last possibility cannot be totally ruled out. *P. psychrophila* grows in cold conditions [39,40], unusual in the natural tropical Amazonian environment; they could be an opportunistic colonizer during the storage in cold rooms previous to selling in the market. Finally, *P. syringae* is a plant pathogen which can infect a wide range of plant species; more than any mineral or other organism is responsible for the surface frost damage in plants exposed to the environment [41]; like *P. psychrophila* tends to be favored by wet and cool conditions [41], being more probable that appear like an opportunistic colonizer during the storage in cool rooms.

**Table 2:** Characteristics of the *Pseudomonas* species found from commercial Amazonian fish and potential risk for humans.

*Pseudomonas* species	Characteristics	Pathogenesis reported for humans
*P. fluorescens*	Opportunistic pathogen in fish [42]	Oncology patients [43]
*P. fragi*	Seafood spoilage [6,44]	No published data about this
	Opportunistic microbiota [6]	
*P. psychrophila*		No *
*P. putida*	Seafood spoilage [44]	Immunodepressed patients [45]
	Cosmopolitan opportunist [46]	Nosocomial infections [47]
*P. syringae*		No *

* They cannot survive at temperatures above 32 °C [39,41], and therefore cannot grow in humans where normal body temperature is 37 °C.

From the phylogenetic point of view, the tree obtained grouped the identified species consistently with previous phylogenetic studies of the genus [47,48]. The same marker, 16S rRNA gene, was used together with other three genes, since although this is a powerful tool for genus assignments, it does not discriminate sufficiently at the inter-species level [49]. In this case the discrimination level of 16S rRNA gene is enough to determine the contamination present in fishes with different species of *Pseudomonas*.

Although we have not found the most dangerous species, the *Pseudomonas* found in our study could be potentially harmful for vulnerable or immunodepressed consumers (Table 2). Infections by *P. fluorescens* and *P. putida* had been reported in old studies [43,44,45,50] (and references therein), and were confirmed later. *P. fluorescens* is a potential pathogen due to their capacity of adhesion to nerves [50], and outbreaks in oncology patients have been discovered [43]. On the other hand, *P. putida* bacteremia seemed to be infrequent and affect mainly immunocompromised patients, with a good prognosis since most cases were cured [45]; however, recent emergent multidrug-resistant and carbapenem-resistant. P. putida isolates cause difficult-to-treat nosocomial infections in seriously ill patients [51]. In brief, these species could cause problems in vulnerable people and do not represent a serious threat for healthy consumers [52], but using a precautionary approach it could be wise to start considering them for future seafood tests. The presence of these pathogens in the products tested here does not mean that they are a risk for consumers; in general *Pseudomonas* sp. represents a hazard for the health when its number exceeds 10^6^–10^7^ CFU/g of product [11,12,15,17] but CFU has not been quantified here. Rather these results could be considered an exploratory work on presence/absence of *Pseudomonas*. If routine surveys were undertaken they should include quantification of the bacteria concentration. RT-PCR based methods could be employed since they can estimate the number of DNA molecules present in a sample. These methods are relatively cheap nowadays and the sequencing cost per sample in our study was approximately 3€ (real cost). However, the analysis of foodborne bacteria is being revolutionized with new sequencing technologies such as NGS [53], and prospects are of better prices for large-scale analysis. On the other hand, this type of PCR-based methods allow to detecting extremely low number of microorganisms based on the production of specific gene copies of a microorganism in question, but it does not distinguish living bacteria from dead cells. Since PCR methodology is rapid (a few hours), additional tests based on the count of total viable microorganisms could be used after initial detection and identification by PCR. Examples are Standard Plate Count [54], determination of most probable number of viable bacteria [55], methods based on fluorescence techniques [56] or direct counting at the microscope [57].

## 4. Conclusions

The possible presence of *Pseudomonas* in fish and seafood should be considered when food imports arrive from countries or areas with *Pseudomonas* endemism and high prevalence of enterotoxigenic-derived diseases. We suggest that routine tests for *Pseudomonas* could be included in the battery of tests aimed at controlling the bacteriological quality of imported fish. PCR-based methodologies, like those employed in this study, are easy and fast and could be considered as a complementary tool to bacterial cultivation.

## References

[1] Saint-Paul U., Zuanon J., Villacorta-Correa M.A., García M., Fabré N.N., Berger U., Junk W.J. (2000). Fish communities in central amazonian white- and blackwater floodplains. Environ. Biol. Fishes.

[2] Fernandes C.C., Podos J., Lundberg J.G. (2004). Amazonian ecology: Tributaries enhance the diversity of electric fishes. Science.

[3] Ardura A., Pola I.G., Linde A.R., Garcia-Vazquez E. (2010). DNA-based methods for species authentication of Amazonian commercial fish. Food Res. Int..

[4] FAO/WHO: Food and Agriculture Organization of the United Nations/ World Health Organization (2009). Microbiological Risk Assessment Series. Risk Characterization of Microbiological Hazards in Food. Guidelines.

[5] Sakata T., Austin B., Austin D.A. (1989). Microflora of Healthy Animals. Methods for the Microbiological Examination of Fish and Shellfish Chichester.

[6] Gram L., Huss H.H., Lund B.M., Baird-Parker A.C., Gould G.W. (2000). Fresh and Processed Fish and Shellfish. The Microbiological Safety and Quality of Foods.

[7] Gram L., Ravn L., Rasch M., Bruhn J.B., Christensen A.B., Givskov M. (2002). Food spoilage-interactions between food spoilage bacteria. Int. J. Food Microbiol..

[8] Nyenje M.E., Odjadjare C.E., Odjadjare L., Tanih N.F., Green E., Ndip R.N. (2012). Foodborne pathogens recovered from ready-to-eat foods from roadside cafeterias and retail outlets in Alice, Eastern Cape Province, South Africa: Public health implications. Int. J. Environ. Res. Public Health.

[9] Bagshaw S.M., Laupland K.B. (2006). Epidemiology of intensive care unit-acquired urinary tract infections. Curr. Opin. Infect. Dis..

[10] Zilberberg M.D., Shorr A.F. (2009). Epidemiology of healthcare-associated pneumonia (HCAP). Semin. Respir. Crit. Care Med..

[11] Mena K.D., Gerba C.P. (2009). Risk assessment of *Pseudomonas aeruginosa* in water. Rev. Environ. Contam. Toxicol..

[12] Jiwa S.F.H., Krovacek K., Wadstrom T. (1981). Enterotoxigenic bacteria in food and water from an ethiopian community. Appl. Environ. Microbiol..

[13] Jertborn M., Svennerholm A.M. (1991). Enterotoxin-producing bacteria isolated from Swedish travellers with diarrhoea. Scand. J. Infect. Dis..

[14] Bockemühl J., Fleischer K., Bednarek I. (1983). A cholera-like illness in a traveller due to a mixed infection with enterotoxigenic *Escherichia coli*, *Vibrio parahaemolyticus* and *Pseudomonas aeruginosa*. Infection.

[15] Adlard P.A., Kirov S.M., Sanderson K., Cox G.E. (1998). *Pseudomonas aeruginosa* as a cause of infectious diarrhoea. Epidemiol. Infect..

[16] Wong S., Street D., Delgado S.I., Klontz K.C. (2000). Recalls of foods and cosmetics due to microbial contamination reported to the U.S. Food and Drug Administration. J. Food Prot..

[17] Craun G.F., Brunkard J.M., Yoder J.S., Roberts V.A., Carpenter J., Wade T., Calderon R.L., Roberts J.M., Beach M.J., Roy S.L. (2010). Causes of outbreaks associated with drinking water in the United States from 1971 to 2006. Clin. Microbiol. Rev..

[18] (2013). Product Inspection of Imported Fish. Csnsdian Food Inspection Agency. http://www.inspection.gc.ca/english/fssa/fispoi/import/pol/procprode.shtml.

[19] (2011). Microbiological Criteria. Health and Consumers. European Commission. http://ec.europa.eu/food/food/biosafety/salmonella/microbio_en.htm.

[20] Food Safety and Inspection Service. United States Department of Agriculture. http://www.fsis.usda.gov/.

[21] Bennett A.R., Greenwood D., Tennant C., Banks J.G., Betts R.P. (1998). Rapid and definitive detection of *Salmonella* in foods by PCR. Lett. Appl. Microbiol..

[22] Wang R.-F., Cao W.W., Cerniglia C.E. (1997). A universal protocol for PCR detection of 13 species of foodborne pathogens in foods. J. Appl. Microbiol..

[23] Estoup A., Largiader C.R., Perrot E., Chourrout D. (1996). Rapid one-tube DNA extraction for reliable PCR detection of fish polymorphic markers and transgenes. Mol. Mar. Biol. Biotechnol..

[24] Spilker T., Coenye T., Vandamme P., LiPuma J.J. (2004). PCR-based assay for differentiation of *Pseudomonas aeruginosa* from other *Pseudomonas* species recovered from cystic fibrosis patients. J. Clin. Microbiol..

[25] Hall T.A. (1999). BioEdit: A user-friendly biological sequence alignment editor and analysis program for Windows 95/98/NT. Nucleic Acids Symp. Ser..

[26] Thompson J.D., Higgins D.G., Gibson T.J. (1994). Clustal-W, Improving the sensitivity of progressive multiple sequence alignment trough sequence weighting, position-specific gap penalties and weight matrix choice. Nucleic Acids Res..

[27] Tamura K., Dudley J., Nei M., Kumar S. (2007). MEGA4: Molecular Evolutionary Genetics Analysis (MEGA) software version 4.0. Mol. Biol. Evolut..

[28] Hebert P., Cywinska A., Ball S., deWaard J. (2003). Biological identification through DNA barcodes. Proc. R. Soc. B: Biol. Sci..

[29] Kumar S., Gadadkar S. (2000). Efficiency of the neighbour-joining method in reconstructing deep and shallow evolutionary relationships in large phylogenies. J. Mol. Evolut..

[30] Posada D. (2008). jModelTest: Phylogenetic model averaging. Mol. Biol. Evolut..

[31] National Center for Biotechnology Information. http://www.ncbi.nlm.nih.gov/.

[32] Nei M. (1987). Molecular Evolutionary Genetics.

[33] Excoffier L., Laval G., Schneider S. (2005). Arlequin (version 3.0): An integrated software package for population genetics data analysis. J. Evol. Bioinform. Online.

[34] Shiose J., Wakabayashi H., Tominaga M., Egusa S. (1974). A report on a disease of cultured carp due to a capsulated *Pseudomonas*. Fish Pathol..

[35] Alderman D.J., Polglase J.L., Holdich D.M., Lowry R.S. (1998). Pathogens, Parasites and Commensals. Freshwater Crayfish—Biology, Management and Exploitation.

[36] Kusuda R., Toyoshima R. (1976). Characteristics of a pathogenic *Pseudomonas* isolated from cultured yellowtail. Fish Pathol..

[37] Uryu Y., Malm O., Thornton I., Payne I., Cleary D. (2001). Mercury contamination of fish and its implications for other wildlife of the Tapajós Basin, Brazilian Amazon. Conserv. Biol..

[38] Altinok I., Kayisa S., Capkin E. (2006). *Pseudomonas putida* infection in rainbow trout. Aquaculture.

[39] Yumoto I, Kusano T., Shingyo T., Nodasaka Y., Matsuyama H., Okuyama H. (2001). Assignment of *Pseudomonas* sp. strain E-3 to *Pseudomonas psychropdomonas* sp. strain E-3 to *Pseudomonas psychrophila* spp. nov., a new facultatively psychrophilic bacterium. Extremophiles.

[40] Morita R.Y. (1975). Psychrophilic bacteria. Bacteriol. Rev..

[41] Hirano S.S., Upper C.D. (1990). Population biology and epidemiology of *Pseudomonas syringae*. Annu. Rev. Phytopathol..

[42] Bruno D.W., Ellis A.E. (1996). Salmonid Disease Management. Dev. Aquac. Fish. Sci..

[43] Hsueh P.R., Teng L.J., Pan H.J., Chen Y.C., Sun C.C., Ho S.W., Luh K.T. (1998). Outbreak of *Pseudomonas fluorescens* bacteremia among oncology patients. J. Clin. Microbiol..

[44] Von Graevenitz A., Weinstein J. (1971). Pathogenic significance of *Pseudomonas fluorescens* and *Pseudomonas putida*. Yale J. Biol. Med..

[45] Yoshino Y., Kitazawa T., Kamimura M., Tatsuno K., Ota Y., Yotsuyanagi H. (2011). *Pseudomonas putida* bacteremia in adult patients: five case reports and a review of the literature. J. Infect. Chemother..

[46] Timmis K.N. (2002). *Pseudomonas putida*: A cosmopolitan opportunist *par* excellence. Environ. Microbiol..

[47] Yamamoto S., Kasai H., Arnold D.L., Jackson R.W., Vivian A., Harayama S. (2000). Phylogeny of the genus *Pseudomonas*: Intrageneric structure reconstructed from the nucleotide sequences of gyrB and rpoD genes. Microbiology.

[48] Franzetti L., Scarpellini M. (2007). Characterisation of *Pseudomonas* spp. isolated from foods. Ann. Microbiol..

[49] Mulet M., Lalucat J., García-Valdés E. (2010). DNA sequence-based analysis of the *Pseudomonas* species. Environ. Microbiol..

[50] Picot L., Mezghani-Abdelmoula S., Merieaua A., Lerouxb P., Cazina L., Orangea N., Feuilloley M.G.J. (2001). *Pseudomonas fluorescens* as a potential pathogen: Adherence to nerve cells. Microbes Infect..

[51] Kim S.E., Park S.H., Park H.B., Park K.H., Kim S.H., Jung S.I., Shin J.H., Jang H.C., Kang S.J. (2012). Nosocomial *Pseudomonas putida* bacteremia: High rates of carbapenem resistance and mortality. Chonnam Med. J..

[52] Gilardi G.L. (1972). Infrequently encountered *Pseudomonas* species causing infection in humans. Ann. Int. Med..

[53] Wilson M.R., Allard M.W., Brown E.W. (2013). The forensic analysis of foodborne bacterial pathogens in the age of whole-genome sequencing. Cladistics.

[54] LeChevallier M.W., Seidler R.J., Evans T.M. (1980). Enumeration and characterization of standard plate count bacteria in chlorinated and raw water supplies. Appl. Environ. Microbiol..

[55] Hihgsmith A.K., Abshire R.L. (1975). Evaluation of most-probable-number technique for the enumeration of *Pseudomonas aeruginosa*. Appl. Microbiol..

[56] Breeuwer P., Abee T. (2000). Assessment of viability of microorganisms employing fluorescence techniques. Int. J. Food Microbiol..

[57] Kogure K., Simidu U., Taga N. (1979). A tentative direct microscopic method for counting living marine bacteria. Can. J. Microbiol..

